# A systematic review of the proposed etiologies of the 2021–2022 outbreaks of pediatric acute hepatitis of unknown etiology

**DOI:** 10.3389/fped.2023.1285348

**Published:** 2023-11-15

**Authors:** Lauren Lewis, Carly van Wylick, Daniel J. Mulder

**Affiliations:** ^1^ Department of Pediatrics, Gastrointestinal Diseases Research Unit, Queen’s University, Kingston, ON, Canada; ^2^ Department of Biomedical and Molecular Sciences, Queen’s University, Kingston, ON, Canada; ^3^ Department of Medicine and the Translational Institute of Medicine, Queen’s University, Kingston, ON, Canada

**Keywords:** disease outbreak, liver failure, emerging communicable diseases, pediatric hepatology, causes of liver disease in children

## Abstract

In April 2022, the World Health Organization (WHO) declared a global outbreak of acute hepatitis of unknown etiology (AHUE) with a high risk of severe outcomes, for which various etiologies have been proposed by the literature. This study examines primary reports of pediatric AHUE cases and summarizes the proposed etiologies. This systematic review collected and evaluated published peer-reviewed articles, official data, and clinical reports of AHUE cases that met the WHO working case definition. 19 hypothesized etiologies for AHUE were identified from 36 sources, which fell into eight categories. While human adenovirus (HAdV) infection, viral infection, and immune-mediated responses were commonly suspected as causes of AHUE, no definitive etiology or epidemiological link has been established. However, recent evidence implicates adeno-associated virus-2 (AAV2) as a likely significant contributor. Conducting a comprehensive literature review following outbreaks is necessary for developing responsive strategies and protocols.

## Introduction

In April 2022, the World Health Organization (WHO) declared an outbreak of acute hepatitis of unknown etiology (AHUE) after clusters of cases appeared throughout the world. A working case definition was developed, identifying affected individuals as previously healthy and under the age of 16, presenting with acute hepatitis (non-A-E) with serum aminotransaminases greater than 500 IU/L (alanine aminotransferase [ALT] or aspartate aminotransferase [AST]), since 1 October 2021. Clinical presentation varies, with most experiencing abdominal pain, diarrhea, and vomiting, followed by severe acute hepatitis, jaundice, and elevated levels of liver enzymes (1). As of November 2022, 1,354 cases have been reported in 40 countries (2, 3). While the viruses involved are commonly found in unaffected populations, this outbreak was associated with a higher risk of severe outcomes including hospitalization and acute liver failure necessitating transplantation (4). Although no clear unifying epidemiologic or etiologic link between cases and clusters had been found, many published studies have postulated about the possible cause of these outbreaks, providing valuable expert opinion that is important to systematically evaluate. Despite a recent decline in reported cases, this outbreak highlights our limited understanding of AHUE and the need for a systematic report of the etiologies supported by the literature to aid future investigations and prevention strategies. This study assesses the published primary reports of pediatric AHUE cases and provides a comprehensive, semi-quantitative summary of the proposed etiologies as reported by those who have observed them. The ultimate goal of this study is to enhance our understanding of potential trends and connections among AHUE cases.

## Methods

This systematic review adhered to the PRISMA guidelines (5). Inclusion criteria included published peer-review articles, data from official websites (i.e., WHO, government, etc.) and clinical reports of AHUE that met the WHO working case definition published between December 2019 and January 2023. Exclusion criteria included cases that did not meet the WHO working case definition, were not peer reviewed or official, not written in the English language, or published before December 2019.

PubMed, Embase, Cochrane Library, Scopus, and Science Direct databases were searched, with dates limited to December 2019 to January 2023. Official websites such as Google Scholar, WHO, European Centre for Disease Prevention and Control, and the Government of Canada were also searched. Backwards citation searching was conducted to identify additional studies by examining the titles of sources in the reference lists of the initial set of sources included in our analysis.

This systematic review utilized a search strategy to retrieve relevant studies from multiple databases, employing a combination of keywords: acute hepatitis of unknown origin in children”, “acute hepatitis of unknown etiology in children”, “pediatric acute hepatitis of unknown origin” and “AHUE in children”.

Two independent reviewers conducted the initial screening process for each record, with at least one reviewer reading each full-text source. Data was collected from identified sources by an independent reviewer using the text, figures, tables, and supplementary materials. To reduce uncertainty, an additional independent reviewer was consulted to confirm the collected data. Any discrepancies among data collectors were resolved through group discussion.

The primary sources were examined, and the proposed etiologies described in each source extracted. Due to the subjective nature of the evaluation, the proposed etiologies were categorized by: (1) precise cause (with no upper limit of categories) with the goal of capturing the breadth of the proposed etiologies, and (2) possible causative mechanism (with an *a priori* goal of limiting the possibilities to a maximum of eight). The objective of this systematic review was to provide a comprehensive semi-quantitative summary of the proposed etiologies of AHUE. The nature of this approach precludes statistical analysis.

## Results

**Figure 1:**
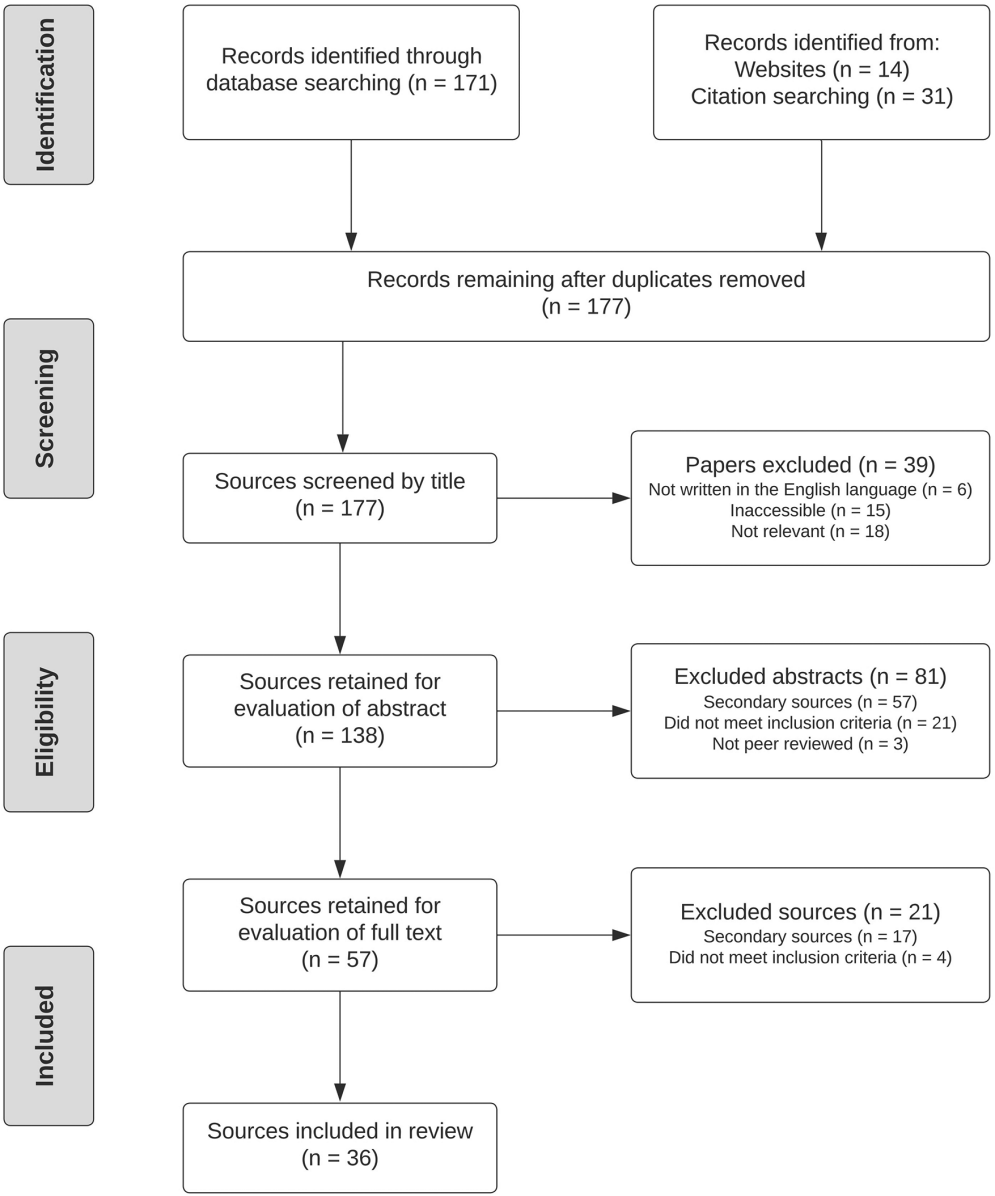
Source inclusion flow diagram based on inclusion and exclusion criteria.

The electronic search process yielded 216 sources relevant for review. After excluding 39 duplicates and studies that did not meet inclusion criteria based on abstract review, 58 sources underwent full text evaluation. Of these, 17 secondary sources and four sources not meeting inclusion criteria were excluded. 36 sources satisfied the inclusion criteria and were included in the data analysis (Figure 1, Supplementary Table S1). It is important to note that studies published by Ho et al. (4), Morofopoulou et al. (6), and Servelitta et al. (7) were published outside of the set time period. However, these articles were accessible in the pre-published format and were therefore included in the study.

**Figure 2:**
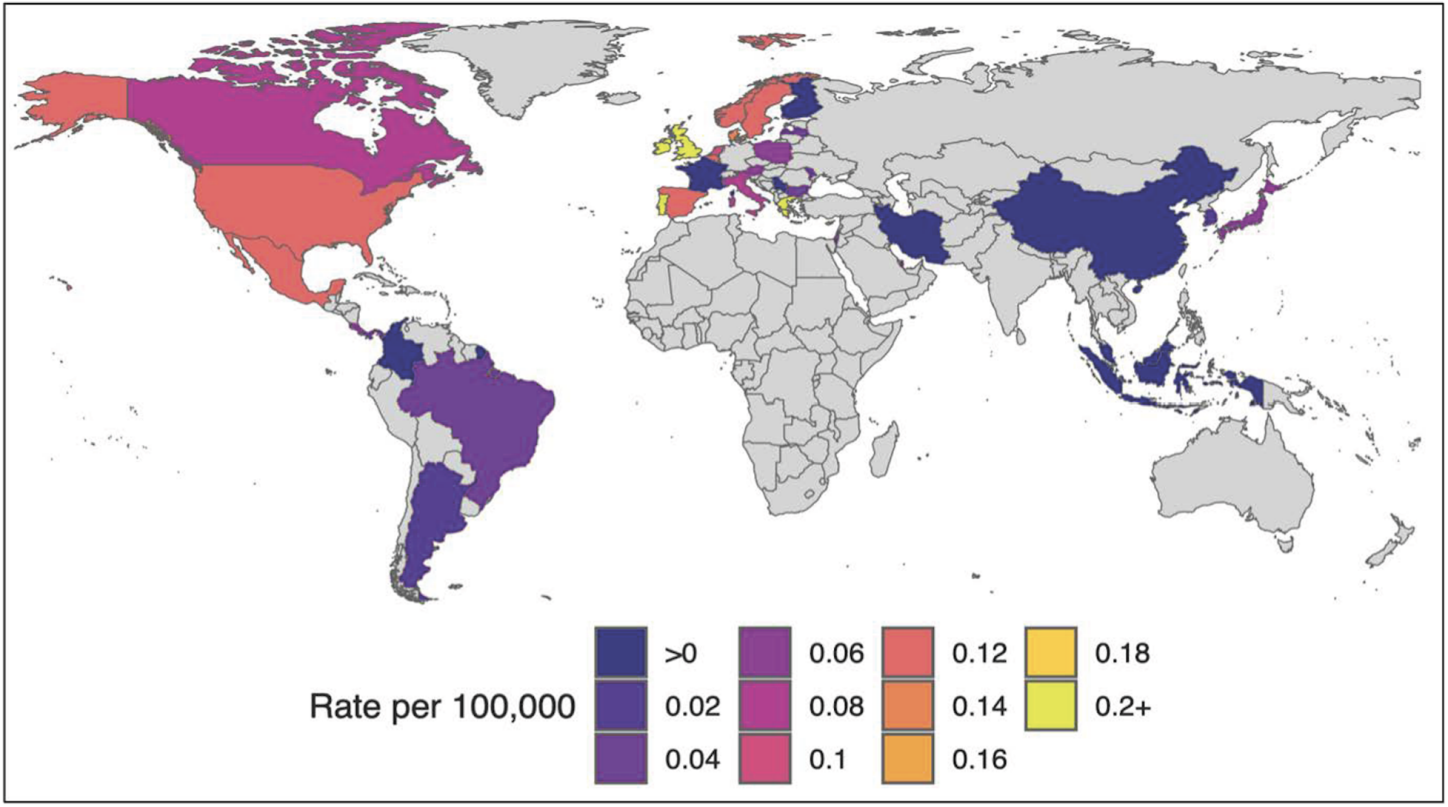
Geographical distribution of the rate per 100,000 population of reported cases of acute hepatitis of unknown etiology.

**Figure 3:**
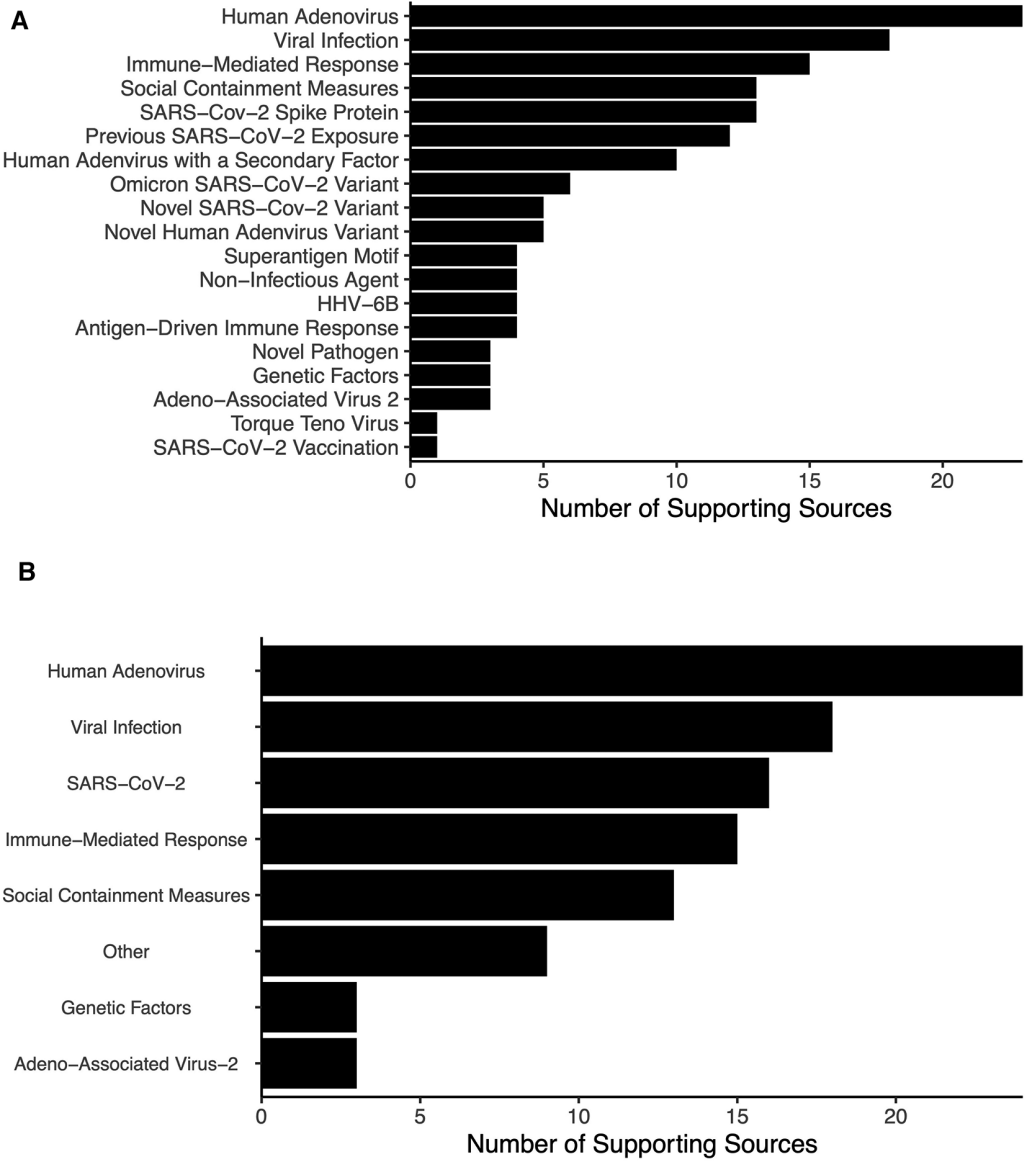
Frequency of the etiologies proposed by sources included in the review, along with the corresponding number of sources categorized by (**A**) precise causes, without any upper limits, and (**B**) possible causative mechanisms with an a priori goal of limiting the causes to a maximum of eight.

Of the included sources, 32 were primary and four were high-quality secondary sources (from governmental or international health organizations). Cases were distributed across the globe (Figure 2). A total of 19 hypothesized etiologies for AHUE were identified (Figure 3A). These etiologies were grouped into eight broader categories: human adenovirus (HAdV)-related, viral infection, severe acute respiratory syndrome coronavirus 2 (SARS-CoV-2)-related, immune-mediated responses, social containment measures, adeno-associated virus-2 (AAV2), genetic factors, and other factors (Figure 3B). The most frequently supported etiologies were HAdV infection, viral infection, immune-mediated response, and social containment measures. SARS-CoV-2 vaccination was the least supported etiology, with only one peer reviewed source. HAdV was the most frequently mentioned viral etiology, with other viruses such as SARS-CoV-2 and AAV2 also proposed. 16 sources hypothesized an etiology related to SARS-CoV-2, including spike protein involvement, previous exposure, the Omicron variant, or a novel variant.

Sources included in the final review were published between January 2022 and January 2023, with most being published between May and July 2022 (Supplementary Figure S1). Reported cases of AHUE mainly came from the WHO-defined Americas and European regions. Analysis of sources providing geographic data revealed a substantial proportion were from the European region (Supplementary Table S2).

## Discussion

This study evaluated 36 sources investigating the proposed causes of the increased rate of AHUE in 2021/2022. 19 etiologies were suggested including HAdV or SARS-CoV-2 infection and immune-mediated responses. While no common epidemiological or mechanistic links among cases have been identified, the abundance of sources suggesting an infectious or immunological etiology implies that a correlation has been observed, even if not yet proven or fully understood. None of the proposed etiologies have been definitively proven, making this summary review of proposed etiologies an excellent starting point for future etiologic investigation.

Studies have investigated whether there has been a statistical increase in reported AHUE cases in 2022. The United States found no increase when analyzing data up to March 2022 (8–11). However, the number of AHUE cases increased significantly in the country after this time (3). Case series of liver evaluations prior to spring 2022, such as a large series of liver biopsies from Germany (10) and Italy (12), do consistently identify cases of acute hepatitis of unknown origin, albeit in rare numbers. It is important to consider that testing for AHUE increased in 2022 following the declaration of the outbreak, and therefore, the increased number of reported cases may be, in part, a result of detection bias.

Social containment interventions during the COVID-19 pandemic reduced the spread of common viruses, leaving children's immune systems vulnerable to routinely circulating viruses. This may have contributed to the emergence of AHUE cases in the first quarter of 2022 as pandemic restrictions were lifted (6, 10, 13). Interestingly, similar “jaundice epidemics”, which could be considered as clusters, similar to those studied herein, occurred after the 1918 influenza pandemic, primarily affecting children (14). The cause was never identified, but it was postulated that social containment measures led to immature pediatric immune systems and increased communicability of the disease (14).

Studies suggest that children are at increased risk of experiencing hepatic injury following SARS-CoV-2 infection. Most children with a prior SARS-CoV-2 infection developed AHUE two to six weeks later, mirroring the timeline of multisystem inflammatory syndrome in children (MIS-C), a complication associated with SARS-CoV-2 infection (15). Liver injury itself is a prominent feature of MIS-C (16). However direct evidence that the pathophysiology of AHUE is related to MIS-C is limited, as the underlying etiology of both conditions is unclear. AHUE is unlikely to be caused by direct SARS-CoV-2 infection, as few cases tested positive on admission in multiple case series (2, 4).

Co-infection of SARS-CoV-2 with an additional virus, such as HAdV, may increase susceptibility to hepatitis, liver failure, and AHUE, especially in children with SARS-CoV-2 viral reservoirs and HAdV infection primarily affecting the gastrointestinal tract (15).

Speculation implicating SARS-CoV-2 mRNA vaccines to AHUE is not supported as most affected children were ineligible for vaccination and those who were eligible had not received the vaccine (1). However, due to the widespread prevalence of SARS-CoV-2 during the AHUE outbreak, it is possible that SARS-CoV-2 detection may be incidental. A case series found that 52% of AHUE cases showed evidence of prior exposure to SARS-CoV-2, a percentage lower than the regional seroprevalence of the virus in the same age group at the time the cases were reported, which ranged from 59% to 72%. Consequently, a direct correlation between SARS-CoV-2 infection and AHUE is unlikely (4).

HAdV has been the most detected pathogen in AHUE samples, suggesting a role in its etiology (17, 18). Studies conducted in Europe found an increase in HAdV DNA concentration in wastewater samples during the peak period of AHUE cases from November 2021 to April 2022 (19, 20). Specifically, HAdV-F40 and HAdV-F41 were detected, which cause acute liver failure and hepatitis in the immunocompromised population (1, 4). While HAdV was not identified in all AHUE cases, it was tested for in various sample types, and sensitivity for detection is highest in whole blood samples, potentially skewing results (17). Histological analyses have not identified HAdV in hepatic cells or tissue, which may be due to sampling error or imply its indirect involvement in AHUE pathogenesis (21).

The epidemiological and clinical features of AHUE suggest an infectious, viral etiology (13). Liver biopsies and electron microscopy failed to detect viral inclusion bodies or particles, posing challenges in establishing an association between a viral agent and pathogenesis (21). It is hypothesized AHUE may be attributed to underlying host factors triggering an abnormal immune response, rather than direct viral infection (15, 18).

Several studies have detected AAV2 in liver and blood samples from AHUE cases, suggesting a role in its etiology (4, 6, 17). High levels of AAV2 vectors used in gene therapies have been associated with hepatotoxicity, caused by an immune response to the AAV2 capsid (22). It is hypothesized that AAV2 may be a biomarker of recent SARS-CoV-2, HAdV, or human herpesvirus-6B (HHV-6B) infection rather than a direct cause of AHUE (4). AAV2 replication is felt to be a satellite virus that requires co-infection with a helper virus, such as HAdV or HHV-6B (4, 6).

Three recent articles have provided strong evidence supporting the role of AAV2 in the pathogenesis of AHUE. Most cases showed elevated levels of AAV2 in both blood and liver samples, with low levels of HAdV, HHV-6B, and Epstein Barr virus detected in some (4, 6, 7). All of viruses were infrequently detected in controls (6, 7). Histological analysis detected AAV2 in ballooned hepatocytes and arterial endothelial cells with prominent T and B cell infiltration, and increased expression of Human Leukocyte Antigen (HLA) class II proteins (4, 6). In summary, AAV2 is a known hepatotoxic virus that is strongly implicated in the pathogenesis of AHUE, likely through co-infection with a helper virus, which may explain the epidemiologic clustering of AHUE cases.

Histological analyses have not detected clear evidence of viral infection, suggesting an indirect, immune-mediated mechanism for liver injury induced by viral infection (4, 6, 15). Histopathological analysis revealed a variable severity of hepatocellular injury, ranging from mild to massive necrosis. No pattern or identifiable cause was identified, likely due to widespread histologic destruction obscuring specific patterns. Several case studies have reported immunohistochemical changes resembling autoimmune hepatitis in liver tissue, even with negative standard autoimmune hepatitis serologies (15). One case, strongly linked to recent SARS-CoV-2 infection, even demonstrated a response to steroid treatment (15). Although this implies that AHUE may be caused by immunological activation, it does not definitively link AHUE to autoimmunity.

Certain Class II HLA alleles, particularly HLA-DRB1*04:01, may increase susceptibility to developing AHUE by promoting antigen-mediated immune responses (4). Samples from AHUE cases have shown positivity for the HLA-DRB1*04:01 allele, suggesting that viral infections, like HAdV or AAV2, may activate a T helper cell-mediated pathological response in genetically susceptible hosts, leading to AHUE (4, 6).

Certain *neuroblastoma amplified sequence* gene variants are known to cause infantile liver failure due to its involvement in Golgi-to-endoplasmic reticulum retrograde transport. Cheng et al. reported a 15-month-old male who presented with AHUE with a homozygous missense mutation in this gene. The exact pathological mechanisms are not fully understood but it is believed to increase susceptibility to fever and inflammation which may contribute to AHUE development (23).

When evaluating differences across health systems, the case identification strategies of are important to consider. Four of the fourteen reported AHUE cases in the Netherlands underwent a liver transplantation, a much higher rate than the 5% of cases necessitating transplantation in the United Kingdom. In the United Kingdom, the National Health System is centralized, coupling AHUE codes with laboratory results, which may result in a higher number of identified relatively milder AHUE cases in the country. In contrast, in the Dutch healthcare system local healthcare providers report cases that meet the case definition, which may result in favouring severe cases that were referred to the pediatric liver transplantation center (18).

Although most clusters of cases occurred in geographically distinct areas, no epidemiological links or common exposures, such as toxins, foods, water sources, international travel, family structure, or pet dogs, have been established (1, 17).

This study was subject to several limitations that merit further discussion. All included sources were written in the English language, which may result in language bias and limit the generalizability of the findings to non-English speaking populations. Additionally, the absence of quantitative data in this study rendered it inherently subjective.

While recent evidence supports the idea of increased clusters rather than higher rates of AHUE, and implicates AAV2 as a potential factor, the exact cause of these clusters remains uncertain, highlighting the need for rapid dissemination of results and stronger international collaborative efforts. It is clear that comprehensive systematic review of the literature is necessary to develop strategies and protocols to react promptly and appropriately to such outbreaks in the future.

Based on our findings, were a similar increase in AHUE clusters to occur, we would propose that the case definition include more broad evaluation (as available). Given the increasing availability of serologic and molecular testing, we would propose that all future cases be evaluated for potential viral markers such as AAV2 and as broad a panel of coronaviruses, in as many tissues as possible.
