# Supplementary material for: A systematic review of the proposed etiologies of the 2021–2022 outbreaks of pediatric acute hepatitis of unknown etiology
Source: Front Pediatr. 2023 Nov 15;11:1285348. doi: 10.3389/fped.2023.1285348 (PMC10715405; doi:10.3389/fped.2023.1285348)
Supplement: Supplementary file 2 [file Datasheet2.pdf]

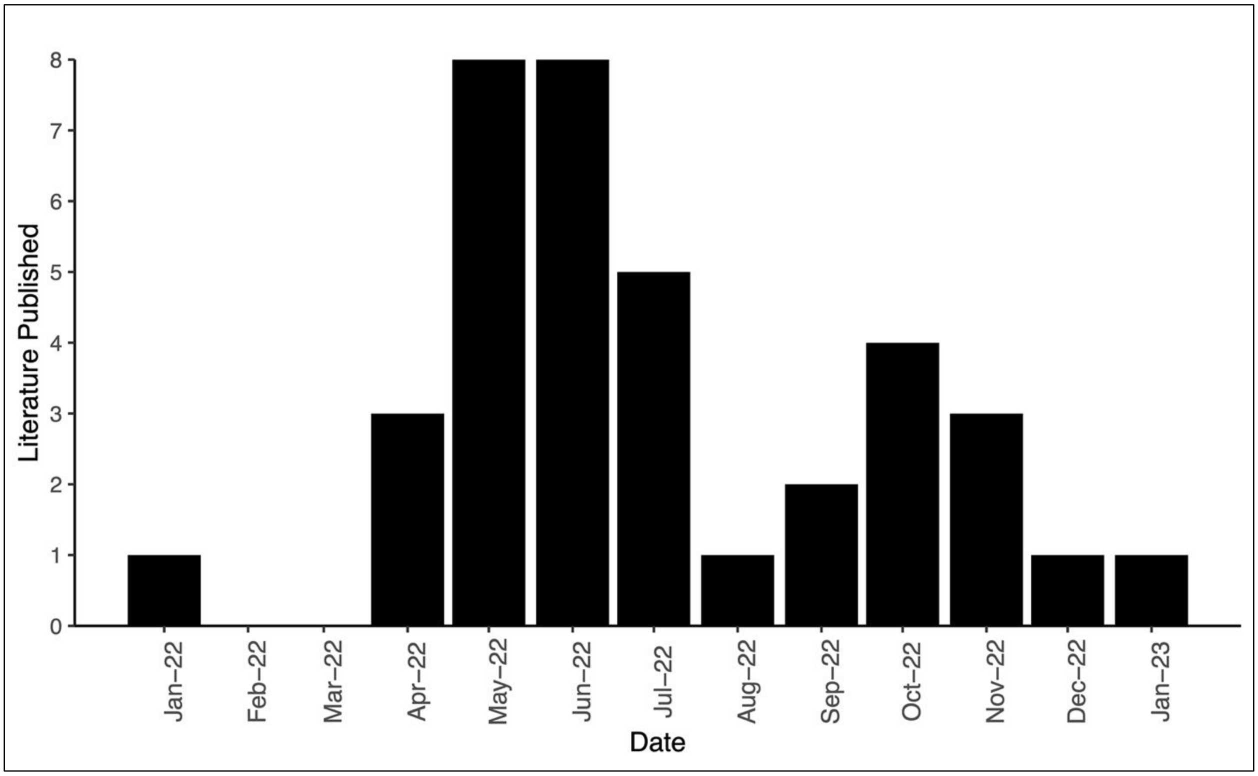

Supplementary Figure 1. Publication timeline for the sources included in the review.

Supplementary Table 1: Characteristics of Included Studies

Source	Reason for Inclusion
European Centre for Disease Prevention and Control, 2022 (1)	Contains data regarding AHUO incidence as well as co-infection in pediatric patients in the European Union
Baker et al., 2022 (2)	Primary data regarding AHUO and adenovirus infection in cases in Alabama
Banc-Husu et al., 2023 (3)	AHUO case series
Brodin & Arditi, 2022 (4)	Investigation of AHUO cases in the United Kingdom
Castro et al., 2022 (5)	Investigation of AHUO cases in Brazil
Cates et al., 2022 (6)	Investigation of AHUO cases in the United States
Chen et al., 2022 (7)	AHUO case report
Cheng et al., 2022 (8)	AHUO case report
Cooper et al., 2022 (9)	AHUO case series
de Kleine et al., 2022 (10)	Investigates whether there has been an increase in AHUO cases in 22 European countries and Israel
Deep et al., 2022 (11)	AHUO case series
Di Dato et al., 2022 (12)	Investigation of AHUO cases in Italy
de Valdoleiros et al., 2022 (13)	Investigation of AHUO cases in Europe
Gutierrez Sanchez et al., 2022 (14)	Case series of AHUO
*Ho et al., 2023 (15)	Investigation of AHUO cases
Kambhampati et al., 2022 (16)	Investigation of AHUO cases in the United States
Kelgeri et al., 2022 (17)	Case series of AHUO in the United Kingdom
Kendall et al., 2022 (18)	Investigation of AHUO cases
Baillie et al., 2022 (19)	Investigates the clinical and epidemiological characteristics of AHUO cases in the United Kingdom
Leiskau et al., 2022 (20)	Investigation of AHUO cases in Germany
Cardoso et al., 2022 (21)	Investigation of AHUO cases in Portugal
Lexmond et al., 2022 (22)	Investigation of cases series of AHUO patients in the Netherlands
Marsh et al., 2022 (23)	Investigation of AHUO cases in Scotland
*Morfopoulou et al., 2023 (24)	Genomic investigation of AHUO cases
Morita et al., 2022 (25)	AHUO case study
Nishiura et al., 2022 (26)	Investigation of AHUO cases
Pellegrinelli et al., 2022 (27)	Investigates whether there has been an increase in adenovirus concentration in wastewater samples in Milan during the period of increased incidence of AHUO cases

Ratho et al., 2022 (28)	Investigates AHUO cases in India
Reyne et al., 2023 (29)	Investigates whether there has been an increase in adenovirus concentration in wastewater in Ireland during the period of increased incidence of AHUO cases.
Rohani et al., 2022 (30)	Investigation of AHUO cases in Iran
Romani Vidal et al., 2022 (31)	Investigation of AHUO cases in Europe
van Beek et al., 2022 (32)	Investigation of AHUO cases in Europe
Verma et al., 2022 (33)	Investigation of AHUO cases
Wollants et al., 2022 (34)	Investigates the whether there has been an increase in adenovirus circulation in the context of the emergence of the increase cases of AHUO in Belgium
Zhou et al., 2022 (35)	AHUO case report
WHO, 2022 (36)	Contains data regarding international AHUO incidence and proposed etiologies
**Servellita et al., 2023 (37)	Investigation of AHUO cases in the United States

*The official publication dates of sources 15 and 24 do not satisfy the inclusion criteria; however, the accepted proofs of these articles were published during the study period and therefore, they are included in the study.

**Does not meet the study's inclusion criteria because of its publication date but has valuable information regarding the underlying etiology of acute hepatitis of unknown origin and is therefore included in the discussion.

Supplementary Table 2: Geographical distribution of the number of reported cases of acute hepatitis of unknown origin and data used by published sources by WHO region.

WHO Region	Number of Reported AHUO Cases	Number of Published Sources
African	0	0
Americas	636	7
South-East Asian	36	1
European	573	22
Eastern Mediterranean	4	2
Western Pacific	105	5

Supplementary Reference List

1 Joint ECDC-WHO Regional Office for Europe Hepatitis of Unknown Origin in Children Surveillance Bulletin. https://www.ecdc.europa.eu/en/hepatitis/joint-hepatitis-unknown-origin-children-surveillance-bulletin. Accessed March 22, 2023, 2023.

2 Baker JM, Buchfellner M, Britt W, et al. Acute hepatitis and adenovirus infection among children—Alabama, October 2021–February 2022. Morbidity and Mortality Weekly Report 2022;71(18):638.

3 Banc-Husu AM, Moulton EA, Shiau H, et al. Acute liver failure and unique challenges of pediatric liver transplantation amidst a worldwide cluster of adenovirus-associated hepatitis. Am J Transplant 2023;23(1):93-100.

4 Brodin P, Arditi M Severe acute hepatitis in children: investigate SARS-CoV-2 superantigens. Lancet Gastroenterol Hepatol 2022;7(7):594-95.

5 Castro R, Ribeiro-Alves M, Veloso VG, et al. Hepatitis of unknown etiology in children in Brazil: A new challenge or the usual scenario ? Braz J Infect Dis 2022;26(6):102715.

6 Cates J, Baker JM, Almendares O, et al. Interim analysis of acute hepatitis of unknown etiology in children aged< 10 years—United States, October 2021–June 2022. 2022.

7 Chen X, Hong J, Li Y, et al. Case report: Severe acute hepatitis in a 22-month-old Chinese boy with Omicron sub-variant BA.2.38. Front Public Health 2022;10(1012638.

8 Cheng Y, Xia Z, Huang C, et al. Case report: A novel cause of acute liver failure in children: A combination of human herpesvirus-6 infection and homozygous mutation in NBAS gene. J Clin Lab Anal 2022;36(5):e24343.

9 Cooper S, Tobar A, Konen O, et al. Long COVID-19 Liver Manifestation in Children. J Pediatr Gastroenterol Nutr 2022;75(3):244-51.

10 de Kleine RH, Lexmond WS, Buescher G, et al. Severe acute hepatitis and acute liver failure of unknown origin in children: a questionnaire-based study within 34 paediatric liver centres in 22 European countries and Israel, April 2022. Euro Surveill 2022;27(19).

11 Deep A, Grammatikopoulos T, Heaton N, et al. Outbreak of hepatitis in children: clinical course of children with acute liver failure admitted to the intensive care unit. Intensive Care Med 2022;48(7):958-62.

12 Di Dato F, Di Giorgio A, Mandato C, et al. Italian children seem to be spared from the mysterious severe acute hepatitis outbreak: A report by SIGENP Acute Hepatitis Group. J Hepatol 2022;77(4):1211-13.

13 Sofia R. de Valdoleiros FC, Nick Beeching, Antonino di Caro, Nicola Petrosillo, Onder Ergonul, Eskild Petersen. Hepatitis in Children - Could it be Alfatoxins? In: E. S. o. C. M. a. I. Diseases ed. ESCMID Emerging Infections Task Force (EITaF): Outbreak News. 2022.

14 Gutierrez Sanchez LH, Shiau H, Baker JM, et al. A Case Series of Children with Acute Hepatitis and Human Adenovirus Infection. N Engl J Med 2022;387(7):620-30.

15 Ho A, Orton R, Tayler R, et al. Adeno-associated virus 2 infection in children with non-A-E hepatitis. Nature 2023;617(7961):555-63.

16 Kambhampati AK Trends in acute hepatitis of unspecified etiology and adenovirus stool testing results in children—United States, 2017–2022. MMWR. Morbidity and Mortality Weekly Report 2022;71(

17 Kelgeri C, Couper M, Gupte GL, et al. Clinical Spectrum of Children with Acute Hepatitis of Unknown Cause. N Engl J Med 2022;387(7):611-19.
18 Kendall EK, Olaker VR, Kaelber DC, et al. 2022.
19 Kenneth Baillie JB, Renu Bindra, et al. Investigation into acute hepatitis of unknown aetiology in children in England. United Kingdom: UK Health Security Agency; 2022.
20 Leiskau C, Tsaka S, Meyer-Ruhnke L, et al. Acute severe non-A-E-hepatitis of unknown origin in children - A 30-year retrospective observational study from north-west Germany. J Hepatol 2023;78(5):971-78.
21 Leonor Cardoso MVM, Sofia Lima, Cristiana Carvalho, Arminda Jorge A Case Report of Acute Hepatitis of Unknown Origin. Acta Medica Portuguesa 2022;35(9).
22 Lexmond WS, de Meijer VE, Scheenstra R, et al. Indeterminate pediatric acute liver failure: Clinical characteristics of a temporal cluster of five children in the Netherlands in the spring of 2022. United European Gastroenterol J 2022;10(8):795-804.
23 Marsh K, Tayler R, Pollock L, et al. Investigation into cases of hepatitis of unknown aetiology among young children, Scotland, 1 January 2022 to 12 April 2022. Euro Surveill 2022;27(15).
24 Morfopoulou S, Buddle S, Torres Montaguth OE, et al. Genomic investigations of unexplained acute hepatitis in children. Nature 2023;617(7961):564-73.
25 Morita A, Imagawa K, Asayama K, et al. Immunological characteristics of severe acute hepatitis of unknown origin in a child post SARS-CoV-2 infection. Clinical Immunology 2022;245(109138.
26 Nishiura H, Jung SM, Hayashi K High population burden of Omicron variant (B.1.1.529) is associated with the emergence of severe hepatitis of unknown etiology in children. Int J Infect Dis 2022;122(30-32.
27 Pellegrinelli L, Uceda Renteria SC, Ceriotti F, et al. Wastewater Surveillance Captured an Increase in Adenovirus Circulation in Milan (Italy) during the First Quarter of 2022. Viruses 2022;14(11).
28 Ratho RK, Asati AA, Mishra N, et al. COVID-19 Associated Hepatitis in Children (CAH-C) during the second wave of SARS-CoV-2 infections in Central India: Is it a complication or transient phenomenon. medRxiv 2021:2021.07. 23.21260716.
29 Reyne MI, Allen DM, Levickas A, et al. Detection of human adenovirus F41 in wastewater and its relationship to clinical cases of acute hepatitis of unknown aetiology. Sci Total Environ 2023;857(Pt 2):159579.
30 Rohani P, Sohouli MH, Ezoddin N, et al. Evaluation of Acute Severe Hepatitis of Unknown Origin in Three Children in Iran: A case series. Iranian Journal of Pediatrics 2022;32(3).
31 Romani Vidal A, Vaughan A, Innocenti F, et al. Hepatitis of unknown aetiology in children - epidemiological overview of cases reported in Europe, 1 January to 16 June 2022. Euro Surveill 2022;27(31).
32 van Beek J, Fraaij P, Giaquinto C, et al. Case numbers of acute hepatitis of unknown aetiology among children in 24 countries up to 18 April 2022 compared to the previous 5 years. Euro Surveill 2022;27(19).
33 Verma A, Vimalesvaran S, Lampejo T, et al. Use of cidofovir in recent outbreak of adenovirus-associated acute liver failure in children. Lancet Gastroenterol Hepatol 2022;7(8):700-02.

34 Wollants E, Keyaerts E, Cuypers L, et al. Environmental circulation of adenovirus 40/41 and SARS-CoV-2 in the context of the emergence of acute hepatitis of unknown origin. medRxiv 2022:2022.06. 08.22276091.
35 Zhou YJ, Gu HY, Tang QQ, et al. Case report: A case of severe acute hepatitis of unknown origin. Front Pediatr 2022;10(975628.
36 Multi-Country – Acute, severe hepatitis of unknown origin in children. https://www.who.int/emergencies/disease-outbreak-news/item/2022-DON376.
37 Servellita V, Sotomayor Gonzalez A, Lamson DM, et al. Adeno-associated virus type 2 in US children with acute severe hepatitis. Nature 2023;617(7961):574-80.
